# Owned-Dog Demographics, Ownership Dynamics, and Attitudes across Three States of India

**DOI:** 10.3390/ani14101464

**Published:** 2024-05-14

**Authors:** George Brill, Amit Chaudhari, Katherine Polak, Suchitra Rawat, Divyanshi Pandey, Pooja Bhatt, Parul Kevin Dholakia, Anju Murali

**Affiliations:** Humane Society International, 1255 23rd Street NW, Suite 450, Washington, DC 20037, USA

**Keywords:** dog ownership, animal welfare, India, pet demographics, public health

## Abstract

**Simple Summary:**

This study examined patterns of dog ownership in three different states in India. The main aim was to understand who owns dogs, their reasons for owning them, and how these patterns vary in different areas. We conducted quantitative surveys to gather information on the number of people who own dogs, their backgrounds, and their attitudes towards their pets. Our findings show that there are significant differences in dog ownership depending on where people live and their economic status. In particular, we found settlements in Gujarat state to possess significantly fewer privately owned dogs that Tamil Nadu and Uttarakhand. These results are important because they help us understand the needs of dog owners and their pets in different parts of India. This information can be used by those making decisions about public health and animal welfare, such as how to manage stray dogs or prevent diseases that can spread from dogs to humans. This study is valuable as it provides a clearer picture of dog ownership in India, which can help improve the lives of both people and animals.

**Abstract:**

This paper presents the demographics, dynamics, and attitudes of dog ownership across three states in India. The background of this research is set against the increasing significance of pet ownership in urban Indian contexts, with a particular focus on understanding the variations in dog-ownership patterns and their implications for public health and animal welfare. We employed a survey-based approach, gathering quantitative survey data from dog owners (*n* = 563) and non-dog-owners (*n* = 9282) across different socioeconomic and geographic backgrounds in seven Indian settlements. The results reveal notable differences in dog-ownership patterns, influenced by regional state. In particular, settlements in Gujarat were found to have significantly fewer dog-owning households than those in Tamil Nadu, with no differences found according to settlement size. Dog ownership was found to be more common in households of higher socioeconomic standing, and settlements in Uttarakhand were found more frequently to possess dogs for reasons other than companionship. Data from Ahmedabad and Vadodara, specifically, also indicate rapidly increasing rates of pet ownership. Sterilisation and rabies vaccination proportions were typically low and high, respectively, across all settlements, with few significant differences found among settlements. Confinement of owned dogs at night was significantly lower in Nainital than all other settlements. Differences in attitudes towards roaming dogs between dog owners and non-dog-owners were also examined, with the results indicating both positive and negative trends accordingly. Our results emphasise the need for region-specific strategies in public health and animal welfare policies, acknowledging the diverse nature of pet ownership in India. This research provides valuable insight for policymakers and animal welfare organisations, underlining the importance of tailored approaches to address the unique challenges and opportunities in the Indian context.

## 1. Introduction

Dogs are synonymous with human settlements worldwide, often being classified as owned, community owned, or un-owned according to the nature of their relationship with the human population and their relative confinement by people [1]; the specific terminology and categorisations employed often vary by country and community. In India, specifically, dogs play a unique role in the community, frequently viewed as devoted guardians of peoples’ homes and families [2]. In India, there are an estimated 62 million dogs living on the street [3]. While this population has been well documented, the number of owned dogs in India is not clear. Accurate estimates are important to understand given concerns about dog welfare, breeding standards, pet shop regulations, and public safety, as well as in regard to the unknown and seemingly region-specific degree to which the owned-dog population may be counted among or directly contribute to the street dog population, whether due to the free roaming of pets, abandonment, or interbreeding. Despite India possessing the most significant rabies burden worldwide [4], with most cases being canine-mediated, relatively little attention has been paid to quantifying the total dog population and the dynamics among the various dog populations in the country [5].

### 1.1. Estimating the Owned-Dog Population of India

Home to one of the largest street dog populations in the world, estimates for Indian street dogs are as high as 62 million [3]. However, India is also home to a sizeable owned-dog population, living alongside and interbreeding with their street conspecifics. As is the case for street dogs, owned-dog population estimates for India vary significantly: the lack of a centralised database or registry of dog ownership, in addition to India’s vast and diverse geography, makes it difficult to ascertaining the true size of the population. Organisations have estimated an owned-dog population ranging from 19.5 million, in 2018, which was forecasted to rise to over 31 million by the end of 2023 [6], to as low as 10.2 million [7].

Many Indian settlements exhibit significant overlap and fluidity between free-roaming and owned-dog populations, further complicating population assessments. Indeed, many free-roaming street dogs are privately or community owned, receiving care and support to various extents [8], highlighting the complex nature of dog ownership in India. Such a blurred population delineation necessitates a nuanced understanding of the interplay between human communities and dog populations.

Furthermore, the lack of information on the development and shifts in dog ownership in India makes it difficult to accurately project population estimates for the future. Undoubtedly, however, pet ownership in India is increasing, with preliminary predictions of as much as a 12% growth in the owned-pet population annually [9]; however, in-depth analyses are yet to be conducted. Additional studies throughout India—both rural and urban—are needed to better comprehend the current state and future forecast of the owned-canine population.

The quantification of the total dog population is a crucial metric for understanding the canine demographics of a given region. However, for the purposes of extrapolating and comparing owned-dog populations among geographical regions within India, measures of density represent a standardised (and simpler to obtain) means of assessing the comparative prevalence of owned dogs, both with respect to area and the cohabiting human population. In doing so, there is an opportunity to accurately measure changes in the dog population over time, independent of spatial or other settlement parameter fluctuations, and by which to compare regional densities. Density represents a more useful and contextual metric than the total dog population of an arbitrarily defined and temporally dynamic regional area. Such units have been used previously by the Humane Society International (HSI) in dog population surveys across Asia, Africa, and Latin America (e.g., [10]), and allow for both regional and international comparison.

### 1.2. Public Health Concerns

According to the World Health Organization, India accounts for approximately one-third of all human rabies deaths worldwide [11]. Dog bite injury itself is also a notable concern. A 2011 study by the WHO estimated 17.4 million dog bites are reported annually in India [12]. It is also likely that decreasing trends may simply represent a lack of incident reporting to the relevant authorities [13]. Region-specific surveys present figures as high as 6% of individuals experiencing dog bites over the course of a year, many of which are from pets [14]. Other studies found similarly high rates of owned-dog bites, representing as much as 43.8% and 11.8% of the total dog bite cases in Tamil Nadu and Bhuj, Gujarat, respectively [15,16]. Unfortunately, the dog bite records of government hospitals do not classify the animals responsible as owned or unowned, and there are, as of yet, few studies that have addressed the matter. Accurately determining dog-ownership status is challenging: many owned animals do not possess a visible tag or collar, microchipping is not yet common in India, and it is often impossible to track down respective owners. Indeed, discrepancies in population-specific dog bite cases among different regions of India could be attributed, in part, to differences in pet ownership and management practices.

### 1.3. Current Challenges with Privately Owned Dogs in India

The Animal Welfare Board of India (AWBI) plays a crucial role in ensuring the rights, protection, and welfare of both street and owned dogs in India [17]. In terms of owned dogs specifically, the AWBI focuses on responsible pet ownership and cruelty prevention. It promotes initiatives that encourage pet owners to provide adequate care, nutrition, and medical attention to their dogs. The AWBI also emphasises the importance of spaying/neutering owned dogs to prevent overbreeding and the subsequent abandonment of puppies.

The responsibility for appropriate bylaws regarding pet registration and other regulations fall under local authorities in India, with several Indian states and municipalities possessing their own laws and guidelines for pet registration and breeding. For example, the Maharashtra Animal Preservation (Amendment) Act, 2015 [18], requires all pet dogs to be registered with the local authorities and to wear identification tags. The Tamil Nadu government has also issued guidelines for pet breeding and has mandated the registration of pet dogs [19,20]. The Noida, Gurgaon, Bengaluru, and Bhubaneshwar Municipal Corporations also recently announced bylaws on pet dogs (e.g., [21,22,23]).

Despite such measures, however, adherence to regulations and enforcement varies widely by region—only a tenth of household pets in East Delhi, for example, are registered despite it being a legal requirement [24]—and owned dogs in India continue to face a variety of health and welfare challenges. The lack of effective regulations around dog breeding and pet registration in India has led to a proliferation of unscrupulous breeders and pet shops, many of which prioritise profit over the welfare of the animals they sell generating serious consequences for both the animals and their owners.

### 1.4. Purpose of This Study

The aims of this study are twofold. First, in order to better understand the owned-dog population across India as a whole, this study examines questionnaire-sourced demographic data from Indian settlements across three geographically distant states to provide insight into the potential variation in owned-dog population dynamics and density across the country. Second, questionnaire responses regarding the attitudes and practices of dog owners and non-dog-owners towards both owned and free-roaming dogs are explored to better understand the context within which the current issues and trends of the Indian human–dog relationship exist, as well as the variation in such landscapes among states in India. Understanding each of these factors is important in the design of effective management practices, whether focused on owned dogs or street dogs.

## 2. Methods

### 2.1. Study Locations

**Figure 1:**
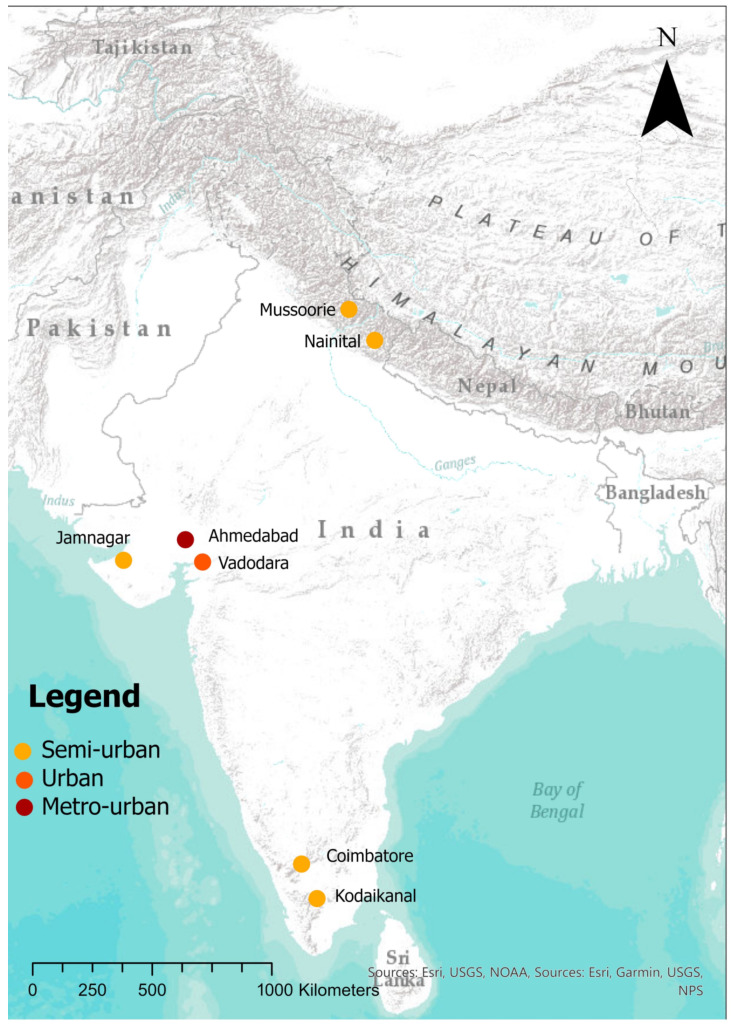
Map of survey locations.

During the period 2013–2021, HSI worked with local governments across India for the implementation and impact tracking of large-scale, city-wide street-dog-population management—or Animal Birth Control (ABC)—programmes. In some of these locations, Knowledge, Attitude, and Practice (KAP) surveys were conducted to better understand the dynamics of private dog ownership. Carried out between 2017 and 2019, they were conducted in the following seven Indian settlements, ranging in size from semi-urban hill stations to some of the largest cities in India (see Figure 1): Jamnagar (population: ~600,000), Ahmedabad (population: ~7,300,000), and Vadodara (population: ~1,800,000) in the State of Gujarat; Mussoorie (population: ~30,000) and Nainital (population: ~40,000) in the State of Uttarakhand; and Coimbatore (population: ~1,900,000) and Kodaikanal (population: ~40,000) in the State of Tamil Nadu (see Supplementary Materials for census data). Together, these represent samples from three states situated in the west, north, and south of India, respectively. Study areas were selected according to areas within which street-dog-population management programs were to be conducted, in all cases coinciding with municipal or town boundaries.

In Gujarat, Ahmedabad (23.0225° N, 72.5714° E) is located along the Sabarmati River, characterised by an arid climate, while Vadodara (22.3072° N, 73.1812° E) lies farther inland, featuring a temperate climate and fertile plains. Jamnagar (22.4707° N, 70.0577° E) is a coastal city along the Arabian Sea. In Tamil Nadu, Coimbatore (11.0168° N, 76.9558° E) is situated in the Western Ghats foothills, with a transition from plains to hilly topography. Kodaikanal (10.2381° N, 77.4892° E) is a hill station surrounded by lush forests and lakes, providing an isolated environment for dog ownership. In Uttarakhand, Nainital (29.3803° N, 79.4636° E) is located in the Kumaon foothills of the Himalayas, near Naini Lake. Mussoorie (30.4591° N, 78.0667° E) is perched on the slopes of the Garhwal Himalayas. Both cities represent mountainous environments, influencing dog-ownership practices.

The socioeconomic context in Gujarat is dominated by the economic activities characteristic of each settlement, as follows: Jamnagar’s reliance on petrochemical industries, Ahmedabad’s textile and garment economy, and Vadodara’s educational and industrial enterprises. The economies of Coimbatore and Kodaikanal, in Tamil Nadu, are predominantly driven by textile and engineering industries and tourism, respectively. Similarly, Nainital and Mussoorie, in Uttarakhand, depend on a tourism-centred economy. It is likely that the tourism-based economic contexts of these three cities are a significant influence on the attitudes towards both owned and roaming dogs. Both settlements in Uttarakhand also possess a focus on ecological conservation efforts.

### 2.2. Household Surveys

Household questionnaires were designed to explore the knowledge, attitudes, and practices surrounding privately owned and free-roaming dogs in the survey regions; see Table S1, Supplementary Materials, for a list of the questions in the questionnaires as matched across settlements. This included questions about the household, respondent, and their dog-ownership status. The attitudes and perceptions of street dogs were then explored via questions on street dog interactions, provisioning of food and water, and concerns. Attitude statements were recorded using a Likert scale. If the respondent claimed ownership of one or more dogs, an integrated secondary survey would automatically be initiated to record each dog’s age, sex, sterilisation status, and rabies vaccination status. Owner perceptions concerning these details were also examined. Where necessary, questionnaires were adapted by survey location to best fit with the needs of the ABC programme. Only the results of select survey questions related to pet ownership are included in this study. All questions generated quantitative data in the form of predetermined answer options, Likert-style scales, or binary responses. Questionnaires were designed in English before translation into the appropriate local languages for each settlement. Survey teams were trained in questionnaire delivery and linguistic translation of questions on a question-by-question basis to ensure consistent data collection. The surveys were conducted over a period of between 1 and 3 weeks depending on the settlement sample size.

Survey teams consisted of a pair of individuals using a smartphone application, Epicollect5 [25], preloaded with the survey questionnaire. Entries could be made on- or offline and were uploaded to a cloud-based database once an internet connection became available. Interviews were conducted along predefined routes designed for street dog surveys (not included in this study) with additional route sections should household density along the survey route be insufficient to achieve the target sample size. Routes were planned in areas spread across each settlement to ensure as representative a demographic distribution as possible. Further, households were selected via systematic random sampling, selecting every ‘n’th household along one side of the street. Only Ahmedabad was surveyed differently, since street surveys were conducted by zone, but census household data were only available on the ward level. Here, wards were stratified using illiteracy rates and human densities reported in the 2011 census [26], and, depending on the ward size, four or eight survey points were randomly selected, around which surveyors walked in zig–zag lines to interview 50 households. Target sample sizes for each route and for the number of routes surveyed within each settlement were determined based upon the goals and targets of the original monitoring and assessment survey for the purpose of informing upcoming street dog management programmes, influenced by resource availability and input from collaborating local municipal bodies. In total, over 9800 households were surveyed across all settlements, with only a very few individuals choosing not to participate (see Table 1 for final sample sizes).

### 2.3. Privately Owned Dog Population and Density Estimates

Estimates for the total number of dog-owning households in each settlement were calculated by multiplying the proportion of dog-owning households in the sample by the total number of households in the settlement. This was multiplied by the number of dogs per dog-owning household in the sample to calculate an estimate of total privately owned dogs in the settlement. Finally, owned-dog density per 100 people was calculated by dividing the total owned-dog population estimate by settlement population size. The total numbers of households and human population figures were sourced from 2011 census data (Government of India 2011) or contemporary municipality estimates, as indicated in Figure 1.

### 2.4. Statistical Analysis

Kendall’s tau was used to test for correlations between the human population and dog density by settlement. Chi-squared tests were used to test for differences in the response proportions among settlements, with pairwise post hoc analyses applied to identify specific patterns. A Bonferroni *p*-value correction for multiple hypothesis testing was applied in each post hoc analysis. Mann–Whitney U tests were used to test for differences in the Likert-style data of the responses between dog owners and non-dog-owners within each settlement, and categories were ranked as ordinal for this purpose in the order they are listed in the figures. The small sample size in Jamnagar is worth noting with regard to the statistical power in pairwise post hoc comparisons involving the settlement.

### 2.5. Data Storage and Analysis

Data were stored in the computers of the principal investigators, cloud-based back-ups, and purpose-built database software, all password protected. Data were only shared with team members as required for data collection and analysis. Statistical analysis was carried out in R version 4.3.3 [27].

## 3. Results

### 3.1. Survey Respondent Demographics

Only a few individuals approached declined participation in the surveys—total sample sizes are indicated in Table 1. The gender split of the survey respondents was approximately equal, with the proportion of female respondents varying between 42.1% (Nainital) and 65.8% (Ahmedabad)—see Table 1. The distribution of the respondents by socioeconomic status varied considerably by settlement (see Table 1)—for example, 66.4% in the upper socioeconomic bracket in Kodaikanal versus only 6.9% in Ahmedabad. Generally, there is a higher proportion of upper-class respondents in settlements located in Tamil Nadu and Uttarakhand than in Gujarat, with respondents in Kodaikanal representing a particularly high majority of detached homeowners.

### 3.2. Privately Owned Dog Population Demography

**Figure 2:**
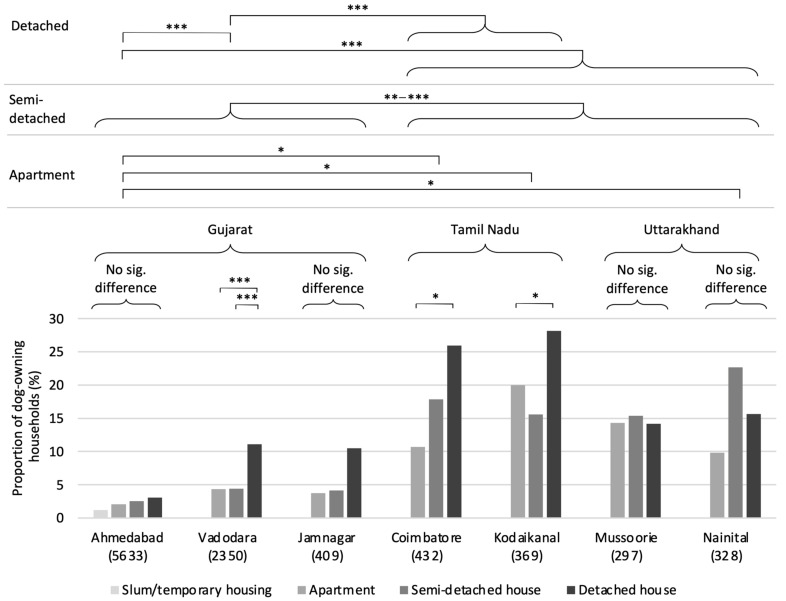
Dog ownership by household type/socioeconomic class; sample sizes in brackets. Values are as the percentage of dog-owning households in each category. Note that for Ahmedabad the categories were defined as ‘slum/temporary housing’, ‘low-income’, middle-income’, and ‘upper-income’, according to the interviewer self-assessment. Post hoc significance levels: * *p* < 0.05; ** *p* < 0.01; *** *p* < 0.001; Bonferroni *p*-value correction applied.

Dog-ownership proportions varied significantly among settlements, with no clear relationship between settlement size (i.e., human population) and owned-dog density per 100 people (*τ* = −0.429, *T* = 6, *p*-value = 0.239); rather, state-wide trends appear to be present. Indeed, pairwise analysis reveals significantly lower proportions of dog-owning households in Gujarat settlements compared to all other settlements in all pairings (*p* < 0.001 in each), while within-state differences are rare, found only between Vadodara and Ahmedabad (*p* < 0.001). No significant differences were found for any of the pairwise comparisons of settlements in Tamil Nadu and Uttarakhand. This pattern is consistent when examined according to different socioeconomic groups, as follows: there are significant differences in dog-ownership proportions among settlements for each socioeconomic bracket with *p* < 0.001 in each case (*X^2^* = 48.683, df = 6; *X^2^* = 103.33, df = 6; *X^2^* = 277.29, df = 6 for apartment, semi-detached, and detached, respectively). The post hoc analysis generally indicates significantly lower dog-ownership proportions in Gujarat (and especially Ahmedabad) compared to Tamil Nadu and Uttarakhand (see Figure 2). While inter-state variance in the proportion of dog-owning households is large—representing a range of approximately ten-fold (0.03–0.06 in Gujarat to 0.27–0.29 in Tamil Nadu; see Table 2)—the average number of dogs owned by dog-owning households was similar across states, with only a few households owning more than a single dog in all settlements (mean ± sd = 1.14 ± 0.14). Using data provided by the 2011 census of India (Government of India 2011) and settlement municipalities, owned-dog densities per 100 people were calculated as 3.21 ± 2.46 (mean ± sd); estimates of total owned-dog populations were also calculated via extrapolation proportional to human population, as presented in Table 2.

### 3.3. Ownership Circumstances

Few significant differences among the dog-ownership proportions were found according to the differing socioeconomic statuses within each settlement. Where differences were found, however, in Vadodara (*X^2^* = 29.147, df = 2, *p* < 0.001), Coimbatore (*X^2^* = 8.663, df = 2, *p* = 0.013), and Kodaikanal (*X^2^* = 6.655, df = 2, *p* = 0.036), households of higher socioeconomic status were found to be more likely to own dogs than those of lower socioeconomic status (see Figure 2). It is possible that the small sample sizes of dog owners, generated by splitting the sample into socioeconomic brackets, inhibit the statistical power necessary to validate the seemingly universal trend of greater ownership in households of higher socioeconomic status, as is apparent in Figure 2.

**Figure 3:**
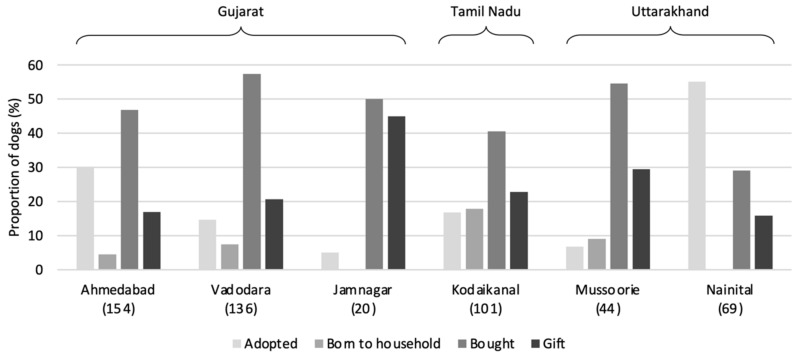
Sources of owned-dog acquisition; sample sizes in brackets. Note also that the vast majority of ‘Adopted’ dogs were adopted from the street; however, a small minority (19 in Ahmedabad and 4 in Vadodara) were adopted from shelters or other people. The number adopted from other sources in other settlements was not clearly specified in response types.

Figure 3 shows that owned dogs were acquired primarily via purchase (typically from unregulated breeders according to qualitative HSI observations) in all settlements, except Nainital, where adoption was much more frequent. It is notable that the vast majority of all adopted dogs were adopted from the street rather than official adoption sources. The practice of giving dogs as gifts was also relatively popular in Jamnagar, and dogs born into households, although still a minority, were far more common in Kodaikanal than elsewhere.

**Figure 4:**
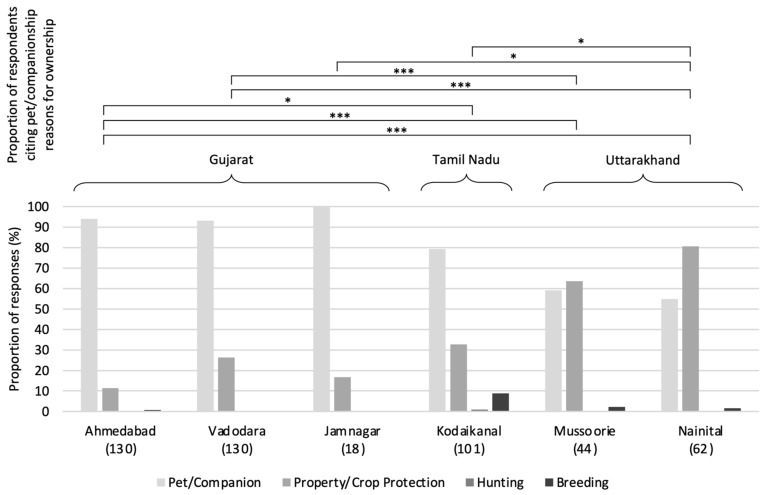
Reasons for dog ownership; sample sizes in brackets. Significance results shown for the pet/companionship response category. Post hoc significance levels: * *p* < 0.05; ** *p* < 0.01; *** *p* < 0.001; Bonferroni *p*-value correction applied.

Dog ownership for the purpose of companionship (Figure 4) was reported in over 50% of responses (respondents could choose multiple options for this question) across all settlements, and represented the most frequent response in Ahmedabad, Jamnagar, Kodaikanal, and Vadodara. Protection purposes were the most frequent response in Mussoorie and Nainital, although companionship remained a close second; dog ownership for hunting and breeding were uncommon across all settlements. Statistically, settlements in Gujarat and Tamil Nadu possessed a greater proportion of respondents citing pet/companionship as a reason for ownership than in Uttarakhand (*X^2^* = 77.027, df = 5, *p* < 0.001); no within-state differences were identified. Regarding reasons given by non-dog-owners for not owning dogs, a lack of need for and a dislike of dogs were the first and second most frequent responses, respectively, across all settlements surveyed (Jamnagar, Mussoorie, Nainital, and Vadodara). Less commonly cited reasons included religious beliefs, an incidental gap in dog ownership, and having no space for a dog.

In Ahmedabad, further questions were asked concerning the duration of dog ownership, with responses indicating that ~75% of owners were first-time dog owners, 46.5% had owned a dog for two years or less, and 23.2% for three to four years. A recent (2023) HSI survey in Vadodara similarly indicates an increase in owned-dog population, reporting an increase from 0.058 to 0.096 dogs per household: an almost doubling of the owned-dog density [HSI, unpublished data].

### 3.4. Ownership Practices

**Figure 5:**
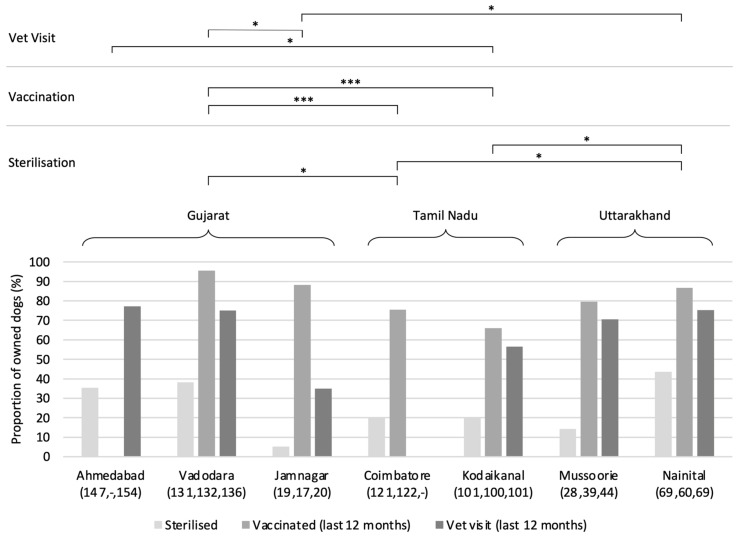
Proportion of owned dogs that are sterilised, vaccinated (last 12 months), and had visited a vet (last 12 months); respective sample sizes in brackets. Note that vaccination and veterinary visits were not investigated in Ahmedabad and Coimbatore, respectively. Post hoc significance levels: * *p* < 0.05; ** *p* < 0.01; *** *p* < 0.001; Bonferroni *p*-value correction applied.

The proportion of owned sterilised dogs was low in all settlements, ranging from ~5% in Jamnagar to just over 40% in Nainital (see Figure 5); only weak differences were found among settlements on post hoc analysis (*X^2^* = 32.21, df = 6, *p*-value < 0.001). The proportions of owned dogs vaccinated for rabies within the last 12 months were greater than 65% in all settlements, in some cases approaching 100%; the post hoc analysis revealed that only Vadodara (Gujarat) possessed statistically greater proportions than settlements in Tamil Nadu (*X^2^* = 37.3, df = 5, *p* < 0.001), although the small sample size for Jamnagar and the lack of data for Ahmedabad may disguise this in fact being a dynamic common to all Gujarat settlements. Veterinary visits during the same year-long period are approximately 10–20% lower than vaccination proportions, with only weak differences in the proportions found among the settlements in the post hoc comparison (*X^2^* = 27.068, df = 5, *p* < 0.001).

When owners of non-sterilised dogs were asked why they had not done so, across all settlements they most frequently reported that it was unnecessary. Further concerns regarding dog safety and a desire for puppies were also raised. Being short of time was frequently given as a reason for a lack of both sterilisation and vaccination in Coimbatore.

**Figure 6:**
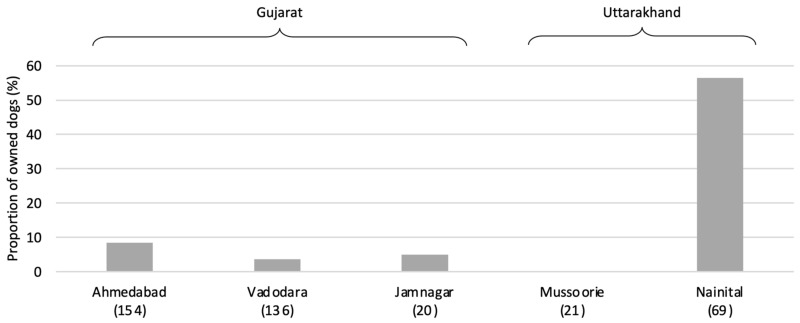
Proportion of owned dogs allowed to roam free at night. Note that dog confinement status was not investigated in Kodaikanal and Coimbatore. Post hoc analysis (with Bonferroni *p*-value correction) indicated a significant difference of at least *p* < 0.01 in all pairwise tests with Nainital.

Confinement practices—the degree to which owned dogs were allowed to roam free (asked regarding overnight practices)—were generally consistent across settlements, with low proportions of owned dogs permitted to roam free at night (see Figure 6). The exception was Nainital, where over half the owned-dog population was reported to be allowed to roam freely, significantly greater than all other settlements in the post hoc analysis (*X^2^* = 120.71, df = 4, *p* < 0.001).

### 3.5. Attitudes of Dog Owners towards Free-Roaming Street Dogs

**Figure 7:**
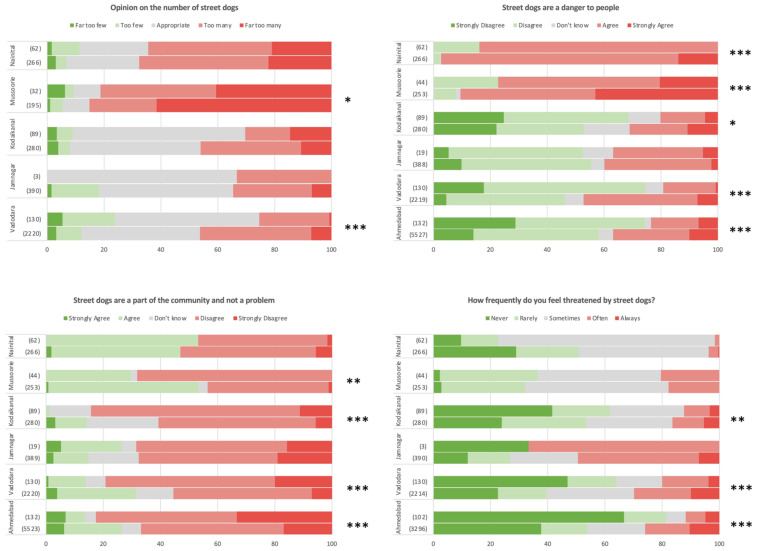
Attitudes of dog owners (**top of each pair**) and non-dog-owners (**bottom of each pair**) towards free-roaming street dogs. Shown as proportion of survey responses. Note small sample size of dog owners in Jamnagar. Significant differences between dog owners and non-dog-owners indicated as * *p* < 0.05; ** *p* < 0.01; *** *p* < 0.001.

Dog-owners were generally more tolerant of street dogs than non-dog-owners, with marginally more positive opinions on the number of roaming street dogs, despite a general consensus in both groups in all settlements examined that street dogs are too many (see Figure 7); only in Vadodara and Mussoorie were there significant differences between dog owners and non-dog-owners regarding opinions on street dog numbers. Dog owners less frequently report street dogs to be a danger to people in all settlements but Jamnagar (note small sample size), and felt less threatened by street dogs than non-dog-owners in Kodaikanal, Vadodara and Ahmedabad. Curiously however, dog owners were less likely to consider street dogs as part of the community and not a problem than non-dog-owners, with significant differences found in Mussoorie, Kodaikanal, Vadodara and Ahmedabad. All such trends are marginal in magnitude, however, with both dog owners and non-dog-owners generally considering free-roaming dogs to be a problem in all cities. Interestingly, opinions on the number of street dogs and them representing a danger are particularly negative in Uttarakhand, despite comparatively infrequent threat perception. These trends are shown in Figure 7.

**Figure 8:**
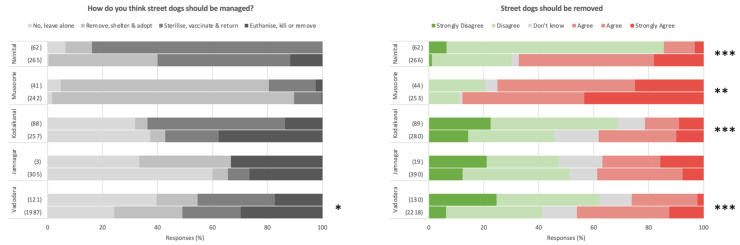
Attitudes of dog owners (**top of each pair**) and non-dog-owners (**bottom of each pair**) towards the management of free-roaming street dogs. Shown as proportion of survey responses. Note small sample size of dog owners in Jamnagar. Significant differences between dog owners and non-dog-owners indicated as * *p* < 0.05; ** *p* < 0.01; *** *p* < 0.001.

**Figure 9:**
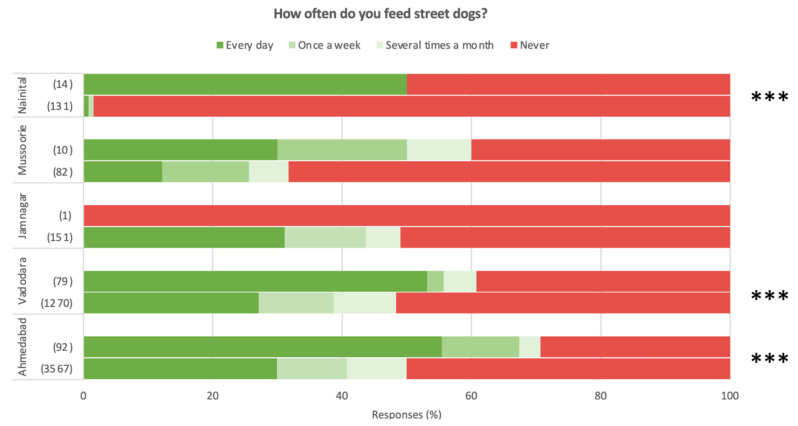
Street dog provisioning frequency by dog owners (**top of each pair**) and non-dog-owners (**bottom of each pair**). Shown as the proportion of survey responses. Note small sample size of dog owners in Jamnagar. Significant differences between dog owners and non-dog-owners indicated as * *p* < 0.05; ** *p* < 0.01; *** *p* < 0.001.

Regarding opinions on street dog management, Figure 8 reveals no clear trends between dog owners and non-dog-owners (except for a weakly significant more aggressive management preference in non-dog-owners than dog owners in Vadodara), however dog owners as a population appear to view the removal of street dogs less favourably than non-dog-owners in all settlements surveyed except Jamnagar (again note the low sample size) when questioned on this management strategy in isolation. Finally, there appears to be a higher frequency of street dog provisioning (feeding) in dog owners than non-dog-owners in each Ahmedabad, Vadodara and Nainital (see Figure 9).

### 3.6. Dog Bite Incidence and Rabies Knowledge

**Figure 10:**
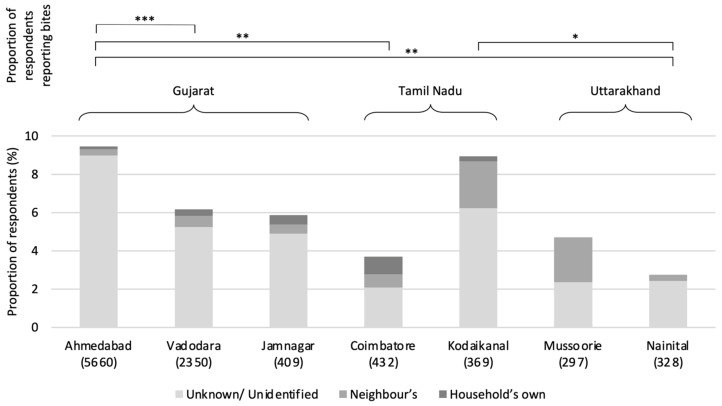
Proportion of respondents reporting dog bites to a member of the household within the last 12 months; sample sizes are in brackets. The ownership statuses of the responsible dogs are indicated. Post hoc significance levels: * *p* < 0.05; ** *p* < 0.01; *** *p* < 0.001; Bonferroni *p*-value correction applied.

With the proportion of households reporting bite incidences over the year prior to the survey varying significantly among settlements from 2 to 10% (*X^2^* = 57.981, df = 6, *p* < 0.001; pairwise analysis indicating particularly high prevalence in Ahmedabad), Figure 10 indicates that the majority of bites were associated with unknown or unidentified dogs. However, owned dogs do contribute to this total, with dogs owned by the household or neighbours representing 30.3% and 43.8% of bites in Kodaikanal and Coimbatore, respectively. In Jamnagar owned-dog bites accounted for 50.0% of total bites, although this was calculated from a total sample of only 14 bites. Only Ahmedabad reported proportions of owned-dog bites lower than 10% (5.1%), with owned-dog bites in Nainital, Vadodara, and Jamnagar representing 11.1%, 15.2%, and 16.2% of incidents, respectively. Knowledge of rabies signs was surveyed in Vadodara and Jamnagar, and, when presented with a list of rabies symptoms as presenting in dogs, 56.7% and 75.3% of respondents respectively identified at least one as a symptom of the disease and 43.4% and 24.8% reported as either not knowing the symptoms or asserted that those presented were wrong.

## 4. Discussion

### 4.1. Owned-Dog Density

Data on owned-dog population dynamics and density are essential for informing dog population management programmes, even in cases where programmes focus primarily on a reduction in the number of free-roaming dogs. Indeed, the data presented here on confinement practices indicate the potential for large and variable degrees of overlap, and even migration (via adoption), between the two populations, with implications as to the requirements for parallel owned-dog management—whether through sterilisation or simply better isolation from free-roaming animals—in order to provide street dog programmes the best chance of reaching their goals.

Estimates of a total owned-dog population are, however, difficult to accurately calculate, and relying on the extrapolation of smaller surveys to entire settlements is fraught with errors surrounding the temporal and spatial congruences of the dog population and census surveys. So too are the use of total population estimates in the comparison of dog population dynamics among settlements, additionally producing values that are somewhat meaningless given the variations in total settlement sizes and precise municipal boundaries. Rather more useful are measures of owned-dog density, which, assuming a representative household sample is used, allow for direct comparisons of owned-dog populations among locations—as in this study—and even internationally [10]. Such figures also provide useful metrics for the tracking of changes over time via follow-up surveys, remaining largely independent of changes in settlement growth and human population flux.

This study reveals a high variability in owned-dog population densities among Indian settlements, with 13.33 ± 9.06% of households owning dogs (mean ± sd), within the range of previous estimates in India [8,24,28]. However, despite reviews of worldwide surveys associating higher rates of dog ownership with rural areas [5], the data presented here indicate no apparent association between dog density and the urbanity of settlements, with the former seeming rather to vary according to state, with settlements in Tamil Nadu and Uttarakhand possessing higher owned-dog densities than those in Gujarat. This may implicate the role of cultural norms and local circumstances—local attitudes, religious beliefs, and space availability, for example—in determining the rates of dog ownership. Indeed, religion and space availability were both reported as reasons for not owning dogs in the surveys conducted. Equally, with the vast majority of non-dog-owners reporting a lack of need or desire for a dog, personal and cultural whims are clearly foundational to local owned-dog densities. Interestingly, analysis of dog ownership by socioeconomic status indicated that dog ownership is low across all classes in Gujarat, indicating that the greater proportion of respondents from higher classes in Uttarakhand and Tamil Nadu settlements is not the causal factor in owned-dog density disparities among settlements. It is clear that state-specific investigations are warranted for a deeper understanding of owned-dog populations in India.

In Ahmedabad specifically—three-quarters of dog owners being first-time dog owners, with the majority having begun ownership in the last four years or even more recently—it appears that dog ownership represents a dramatically increasing trend, supporting previous observations [9]. Indeed, HSI’s 2023 Vadodara survey indicates an almost doubling of the owned-dog density since 2017 [HSI, unpublished data]. Anecdotal observations made during HSI surveys have indicated an increase in dog ownership among middle and higher socioeconomic Indian classes—a dynamic potentially reflected in the typically higher proportion of dog-owning households in higher socioeconomic categories in the survey data presented here. Such temporal changes in dog-ownership dynamics are little studied, and further longitudinal studies are necessary. It may be that such a trend is representative of a shift in attitudes towards dogs in India more generally; the fact that most dogs across all settlements surveyed were bought or adopted and that companionship composes the majority response related to ownership purpose in all but the most rural of surveyed settlements—a dynamic also found elsewhere in India [24]—does, indeed, appear to support a shift from utility to companionship in Indian dog ownership [29]. Should such observations concerning a sharp uptake in dog ownership prove true across India more broadly, the necessity of well thought-out and applied regulations concerning dog ownership becomes ever more pertinent. While current laws require every pet shop and dog-breeding establishment to register with both state and municipal authorities, systematic improvements in the capacity for enforcement and promotion of such laws are required to ensure their efficacy given the increasing demand for companion dogs, and subsequent proliferation in pet sale and breeding establishments. Further, education surrounding, as well as promotion of, adoption rather than purchase could help to simultaneously relieve the pressure on roaming-dog population management efforts.

### 4.2. Ownership Practices

Sterilisation rates for owned dogs in the settlements studied are relatively low (mean ± sd: 22.51 ± 14.32%), far below the >55% critical proportion modelled to reduce or even stabilise breeding dynamics [30]. While the owned-dog population density is likely little affected by breeding dynamics—rather the desire for pets—the implications for a pool of unsterilised animals living in close vicinity to street-dog populations undergoing meticulous reproductive management protocols are significant. The low proportions of owned-dog sterilisation are, perhaps, in part reflective of the generally poor adherence to municipal and organisation pet ownership guidelines and regulations observed elsewhere in India [24], as well as a general lack of access to affordable spay/neuter services. While veterinary visits are comparatively more common in most of the settlements surveyed, most dog owners exhibit a general lack of knowledge about veterinary care and their pet, as indicated by the lack of interest in spay/neuter surgery. In some cases, owners expressed concern about the welfare of their dog undergoing such a procedure.

The rates of owned-dog confinement were high, with the exception of Nainital, with implications for the proportion of animals that may effectively be counted among the free-roaming population, and, thus, the increased interaction between unsterilised owned dogs and true street dog individuals [5,28]—an important factor to consider in the planning, execution, and management of ABC programmes, as well as in the spread of diseases and population-breeding dynamics. The especially high rates of free-roaming owned dogs in Nainital are presumably related to its unique situation in which most dogs are adopted from the street for the purposes of property protection. Both Nainital and Mussoorie exhibit comparatively high levels of dogs owned for protection purposes; these anomalous profiles are likely the result of the unique hill station status of these Uttarakhand settlements: the communities of each are directly or indirectly dependent on tourism, subsequently differing with regard to sociocultural dynamics and ownership practices compared to the settlements of Gujarat and Tamil Nadu. Such findings again emphasise the need for state-specific understanding of dog-ownership and population dynamics.

Indeed, the precise environmental and cultural circumstances of Indian cities have significant influences on dog ownership and owner practices. Mountainous regions, in particular, possess a long cultural history of dog ownership, historically associated with representing the cheapest source of security and protection. This has resulted in a high proportion of street dogs that are, in fact, semi-owned or owned yet allowed to roam freely. Nowhere is this influence more evident than in cities like Nainital and Mussoorie. The challenging mountain terrain of these hill stations, coupled with a history of relying on dogs for guarding and protection, has fostered a unique relationship between the local population and street dogs. As a result, these regions exhibit a more open and communal approach to dog ownership, where street dogs often coexist well with the community.

### 4.3. Attitudes of Dog Owners towards Free-Roaming Street Dogs

Attitudes surrounding street dogs are generally more positive in the present survey compared to the results of previous research [31], with only a minority of respondents, on average, suggesting that street dogs represent a danger to people and most considering there to be appropriate numbers on the streets; the perception of threat was also typically low. However, that a majority across almost all settlements considered street dogs not to be a part of the community indicates a perceptual separation between human and street dog populations, even despite many individuals feeding free-roaming dogs on a regular basis. The comparatively negative attitudes in Uttarakhand—with a majority of respondents asserting that there are too many free-roaming dogs and considering them a danger, as well as lower feeding frequencies and a higher preference for removal—may possibly relate to the effect of street dogs on the towns’ tourism economy.

While the results of this survey indicate little difference between the attitudes of dog owners and non-dog-owners towards street dogs, we see that dog owners tend to be more positive overall, feeding dogs more frequently. This seems logical, given a presumed higher proximity and affinity of dog owners with dogs generally, and matches the results of other studies into pet owner versus non-pet-owner dynamics [31]. Finally, it is interesting to consider attitudes towards street dog in the context that the majority of dogs sourced through adoption originated from the street, again demonstrating the closeness of the human–street dog relationship, and that potential population movement is not only from owned to street via abandonment, but also vice versa via adoption.

While the greater proportion of non-dog-owning respondents from higher socioeconomic classes in Tamil Nadu and Uttarakhand settlements than Gujarat should be acknowledged in the consideration of inter-settlement attitudes and practices towards free-roaming street dogs, no consistent differences in attitudes were found between states.

### 4.4. Dog Bites and Rabies

Dog bites represent a significant public health concern in India, not only regarding injury but also in the context of zoonotic diseases [4,32]; however, very little research has been conducted to establish the part that owned dogs play in bite prevalence across India. Indeed, the source of a dog bite is rarely recorded, and does not appear in hospital records of such incidences. As a result, there is a general assumption that stray dogs are solely responsible, and survey data from East Delhi indicates that 34.2% of survey respondents were unaware of pets as a potential source of rabies, with many pet owners believing that rabies could only originate from stray dogs [24].

In keeping with previous research [8,14], our data demonstrate a high annual dog bite prevalence per household (5.94%), as well as indicating that owned dogs represent a significant contribution to total cases in Coimbatore, Kodiakanal and Jamnagar—a dynamic recognised elsewhere [14,15,16]. Nainital may also possess higher owned-dog bites than reported, if we consider the large proportion of owned dogs allowed to roam free, and the generally high density of owned dogs in the settlement; this too has been stressed in India previously [8].

As such, rabies vaccination programmes must take owned-dog populations into account to reduce overall rabies risk. The percentages of owned dogs vaccinated against rabies in India in previous studies have been low, with a significant majority found to be unvaccinated [8]. Surprisingly, given the relatively low proportions of sterilisation, vaccination proportions in the present sample were consistently high across all settlements surveyed (mean ± sd: 74.8 ± 9.85), even in Jamnagar where veterinary visits were comparatively far lower. Of the six settlements surveyed, all but Kodaikanal (66.0%) achieved the 70% vaccination coverage considered to be effective in rabies control [33]. This is encouraging, although it is possible that interviewees are falsely reporting vaccination on the basis of a perceived surveyor expectation. Most interviewees had some awareness about rabies; however, many individuals surveyed were unaware of the symptoms—this too is in line with previous research indicating similarly high awareness yet poor actual knowledge, as well as a tendency towards lower knowledge in lower socioeconomic regions [34,35]. This is consistent with varied local practices for the understanding and treatment practices of rabies and other zoonotic diseases across India [24,36].

### 4.5. Limitations

A number of limitations are recognised in this study. First, the small sample sizes that result when ownership groups are split by socioeconomic class reduce the power of statistical analysis such that differences in ownership may have become indetectable. Similarly, the small sample size of Jamnagar dog owners is noted as a likely reason for a general lack of statistical differences identified between Jamnagar and settlements of other states, despite consistent differences found regarding the other two settlements of Gujarat: Ahmedabad and Vadodara. In both cases similar trends do appear to be present, yet they cannot be validated with the present sample.

Second, while respondent gender proportions were roughly equal among settlements, and socioeconomic class was examined, additional respondent demographic data such as age were not collected in sufficient detail to examine associated response trends. While it may be assumed that roughly similar age distributions are present across samples from each settlement, this was not validated in the sample.

Third, while the questionnaire employed was developed over many years of pilot assessments and large-scale surveys conducted worldwide by HSI, certain questions may be subject to location-specific biases. For example, it is possible that for questions concerning, for example, vaccination and sterilisation, owners may have answered based on the perceived desired response, rather than the truth. Similarly, no form of resampling methodology was employed to validate questionnaire reliability. Additionally, while interviewers were trained in consistent delivery and, where necessary, translation of the questionnaire, there is minor scope for variation in question delivery by location. However, since questionnaires were not open ended, rather possessing set answer options, the effect is believed to be negligible.

Finally, regarding calculations of dog density and abundance, figures must be considered representative of rough estimates only due to temporal discrepancy between surveys and the 2011 census data. Unfortunately, at the time of publication the 2021 census in India is still yet to be conducted following the COVID-19 pandemic. In a few cases (noted in Table 1), census data were provided at the time of survey, representing more accurate estimations.

## 5. Conclusions

This study presents a descriptive survey of owned-dog populations across seven settlements in three states of India. Generally, it appears that owned-dog population densities and practices are highly variable across settlements, with state-specific dynamics implicating the need for local research in the planning and application of management programmes. Nainital and Mussoorie, popular tourist destinations, possess notably different dynamics than other settlements. Attitudes towards street dogs were generally positive, or at least tolerant, except in Uttarakhand, where responses were more negative; only minor differences between dog owners and non-dog-owners were present. Knowledge concerning owned-dog welfare and adherence to pet ownership guidelines are less than ideal across all settlements, highlighting the need for ongoing educational programmes and promotion of existing regulations across the country. Furthermore, while the percentage of dogs vaccinated was unexpectedly high across all settlements surveyed, a need for greater education surrounding rabies is clear, and, in some locations, a significantly high number of dog bites resulting from pet dogs were reported; conventional assumptions of the sole responsibility of unowned dog in bite and rabies cases are unwarranted, and owned-dog populations must be considered in efforts to address associated public health concerns. Rates of both adoption of street dogs into the owned-dog population, and the degree to which owned-dog populations are allowed to mix with (and, thus, contribute to) street dog populations were also very variable among settlements, indicating the importance of region-specific understanding concerning the significance of the local owned-dog population in affecting breeding dynamics and total population size in the design of street-dog management initiatives.

Together, these results indicate general trends in attitudes towards street dogs and dog ownership that may be tentatively considered representative of urban India more generally: a general tolerance of street dogs, yet desire for a reduced population and the existence of negative interactions on a regular basis; owned dogs typically exhibit low sterilisation proportions despite high proportions of rabies vaccination; and there being generally low levels of comprehensive rabies knowledge. However, and perhaps more significant, our results stress the variability of dynamics between settlements and states, asserting that conclusions drawn concerning dog populations and their reception in one region of India cannot necessarily be extrapolated to another. In particular, our results suggest that differences in economic and social structures of settlements may significantly influence the attitudes and practices surrounding both roaming and owned-dog populations. Finally, our data provide some insight into the recent trend of increasing pet ownership in India, with survey data from Ahmedabad and Vadodara, Gujarat, appearing to indicate a dramatic, recent increase in dog ownership. Such significant growth in the industry further stresses the urgency for greater regulation and attention from local and national authorities. Continued monitoring and enforcement of pet ownership laws and guidelines, as well as education and outreach efforts, are required to promote responsible pet ownership and companion animal welfare in India.

## Figures and Tables

**Table 1:** Demographic distribution of survey respondents by settlement.

Settlement	N	Respondent Gender (% Female)	Socioeconomic Status (%)			
			Slum/Temporary Housing	Low-Income Class/Apartment	Medium Class/Semi-Detached House	Upper Class/Detached House
Ahmedabad	5660	65.8	8.8	35.5	48.8	6.9
Vadodara	2350	58.3	-	26.4	56.4	17.2
Jamnagar	409	62.8	-	25.9	64.8	9.3
Coimbatore	432	55.8	-	17.4	38.9	43.8
Kodaikanal	369	50.4	-	4.1	29.5	66.4
Mussoorie	297	55.2	-	2.4	52.5	45.1
Nainital	328	42.1	-	15.5	59.1	25.3

**Table 2:** Survey details and owned-dog demographics across settlements of three Indian states. Estimates for total owned-dog population and per person density based on the census data of households and human populations from 2011 census (Government of India, 2011); * values were calculated from census estimates provided by municipalities at time of survey. HH = household; DOHH = dog-owning household. Census data used are provided in the Supplementary Materials. Age data were not recorded for Coimbatore.

Settlement	Survey Date	State	Settlement Type	HH Surveyed	DOHH	Owned-Dog Count	Dogs per HH	Dogs per DOHH	HH Owning Dog/s (%)	Total Owned-Dog Population	Dogs per 100 People	Mean Dog Age (Years)	Males per Female
Ahmedabad	Aug 2019	Gujarat	Metro-Urban	5660	132	155	0.03	1.17	2.33	43,041 *	0.59 *	3.98	1.33
Vadodara	Nov 2017	Gujarat	Urban	2350	130	136	0.06	1.04	5.53	21,824	1.25	-	2.27
Jamnagar	Oct 2017	Gujarat	Semi-Urban	409	19	20	0.05	1.05	4.64	5472	0.91	3.65	2.50
Coimbatore	Jul 2017	Tamil Nadu	Urban	432	87	125	0.29	1.44	20.0	82,023	4.34 *	3.05	2.33
Kodaikanal	Jun 2017	Tamil Nadu	Semi-Urban	369	89	101	0.27	1.13	24.12	2584	7.08	2.68	2.00
Mussoorie	Jul 2017	Uttarakhand	Semi-Urban	297	44	44	0.15	1.00	14.9	931	3.09	3.82	1.35
Nainital	Jul 2017	Uttarakhand	Semi-Urban	328	62	69	0.23	1.11	21.8	2155	5.21	3.23	5.26

## Data Availability

All the data collected during the study are stored in both raw and calculated form with the authors (Amit Chaudhari) and can be shared, if needed, for validation. Raw count data are provided in the Supplementary Materials.

## Supplementary Materials

The following supporting information can be downloaded at: https://www.mdpi.com/article/10.3390/ani14101464/s1, Table S1: List of survey questions, Table S2: Census data, Table S3: Count data of socioeconomic variables, Table S4: Count data of dog acquisition sources, Table S5: Count data of reasons for dog ownership, Table S6: Count data of sterilisation, vaccination and vet visits, Table S7: Count data of dogs reported to be free roaming, Table S8: Count data of dog bite incidences.

## References

[1] Taylor L.H., Wallace R.M., Balaram D., Lindenmayer J.M., Eckery D.C., Mutonono-Watkiss B., Parravani E., Nel L.H. (2017). The Role of Dog Population Management in Rabies Elimination—A Review of Current Approaches and Future Opportunities. Front. Vet. Sci..

[2] Subramanian A. (2017). Dogs and the People of India: A Photographic Exploration.

[3] End Pet Homelessness (2020). Index Results—India: State of Pet Homelessness Index. https://stateofpethomelessness.com/the-index/.

[4] Knobel D.L., Cleaveland S., Coleman P.G., Fèvre E.M., Meltzer M.I., Miranda M.E.G., Shaw A., Zinsstag J., Meslin F.X. (2005). Re-evaluating the burden of rabies in Africa and Asia. Bull. World Health Organ..

[5] Davlin S.L., VonVille H.M. (2012). Canine rabies vaccination and domestic dog population characteristics in the developing world: A systematic review. Vaccine.

[6] Minhas A. (2022). India—population of pet dogs 2014–2023 | Statista. https://www.statista.com/statistics/1061130/india-population-of-pet-dogs/.

[7] Walden L. (2015). Worldwide Pet Ownership Statistics | Most Common Pets Around the World—PetSecure. https://www.petsecure.com.au/pet-care/a-guide-to-worldwide-pet-ownership/.

[8] Sudarshan M.K., Mahendra B.J., Madhusudana S.N., Narayana D.A., Rahman A., Rao N.S.N., X-Meslin F., Lobo D., Ravikumar K. (2006). An epidemiological study of animal bites in India: Results of a WHO sponsored national multi-centric rabies survey. J. Commun. Dis..

[9] Tekriwal A. (2022). The New Wave of Pet Parenting Is Driven by Millennials: Tekriwal, Supertail. ET Health World.com 10 January, The Economic Times. https://health.economictimes.indiatimes.com/news/industry/the-new-wave-of-pet-parenting-is-driven-by-millennials-aman-tekriwal-supertails/88763937?redirect=1.

[10] Chaudhari A., Kartal T., Brill G., Amano K.J., Lagayan M.G., Jorca D. (2022). Dog Ecology and Demographics in Several Areas in the Philippines and Its Application to Anti-Rabies Vaccination Programs. Animals.

[11] World Health Organization (2021). Rabies in India. https://www.who.int/india/health-topics/rabies.

[12] Gongal G., Wright A.E. (2011). Human rabies in the WHO Southeast Asia region: Forward steps for elimination. Adv. Prev. Med..

[13] Wani S. (2023). Over 5500 Dog Bite Cases a Day in 2022, Though Lower Than Pre-COVID Count. 6 March Business Standard. https://www.business-standard.com/article/current-affairs/over-5-000-dog-bite-cases-a-day-top-5-states-account-for-60-of-incidents-123030600747_1.html.

[14] Sangeetha S., Sujatha K., William R.F. (2017). An epidemiological study of animal bites among rural population in Tamil Nadu, India. Int. J. Community Med. Public Health.

[15] Charulatha R.J., Umadevi R., Anantha Eashwar V.M. (2021). Pattern of Injuries among Dog Bite Victims in an Urban Area of Kancheepuram District, Tamilnadu. Natl. J. Community Med..

[16] Bharadva N., Mehta S.R., Yerpude P., Jogdand K., Trivedi K.N. (2015). Epidemiology of Animal Bite Cases Attending Tertiary Health Care Centre of Bhuj City of India: A Cross-Sectional Study. Int. J. Interdiscip. Multidiscip. Stud. (IJIMS).

[17] Animal Welfare Board of India (2015). Guidelines—With Respect to Pet & Street Dogs, and Their Care-Givers, and for Residents’ Welfare Associations and Apartment Owners Associations (Report No. 3).

[18] Government of India (2015). Maharashtra Animal Preservation (Amendment) Act. https://www.pcmcindia.gov.in/admin/cms_upload/submission/12778054841366030285.pdf.

[19] Chawla Publications (2016). Tamil Nadu Panchayats (Licensing of Dogs) Rules, 1999. http://www.bareactslive.com/TN/tn860.htm?AspxAutoDetectCookieSupport=1.

[20] Government of Tamil Nadu (2017). Prevention of Cruelty to Animals (Dog Breeding and Marketing) Rules. https://cms.tn.gov.in/sites/default/files/rules/pet_shop_reg_appln_0.pdf.

[21] Bhubaneswar Municipal Corporation (2023). Registration and Proper Control of Dogs) Bye-Laws. https://kalingatv.com/wp-content/uploads/2023/04/Click-here-to-read-Bhubaneswar-Municipal-Corporation-Registration-and-Proper-Control-of-Dogs-Bye-Laws-2023.pdf.

[22] The Indian Express Noida Authority Updates Dog Policy, Releases ‘Dos and Don’ts’. The Indian Express. 12 December 2022. https://indianexpress.com/article/cities/delhi/noida-authority-dog-policy-releases-dos-donts-8320666/.

[23] Debabrata Mohanty (2023). Penalty up to ₹10,000 for Dog Owners in Bhubaneswar If Pet Defecates in Public. Hindustan Times. https://www.hindustantimes.com/cities/others/penalty-up-to-10-000-for-dog-owners-in-bhubaneswar-if-pet-defecates-in-public-101679494826418.html.

[24] Cherian V., Dugg P., Khan A.M. (2020). Prevalence of pet dog ownership in an urban colony of East Delhi and awareness regarding canine zoonotic diseases and responsible pet ownership among dog owners. Indian J. Community Med..

[25] Centre for Genomic Pathogen Surveillance (2023). Epicollect5 [Mobile App]. https://five.epicollect.net.

[26] Government of India (2011). Census of India. [Database]. https://censusindia.gov.in/census.website/data/census-tables.

[27] R Core Team (2024). R: A Language and Environment for Statistical Computing.

[28] Sudarshan M.K., Mahendra B.J., Narayan D.H. (2001). A community survey of dog bites, anti-rabies treatment, rabies and dog population management in Bangalore city. J. Commun. Dis..

[29] Volsche S., Mohan M., Gray P.B., Rangaswamy M. (2019). An exploration of attitudes toward dogs among college students in Bangalore, India. Animals.

[30] Di Nardo A., Candeloro L., Budke C.M., Slater M.R. (2007). Modeling the effect of sterilization rate on owned dog population size in central Italy. Prev. Vet. Med..

[31] Corfmat J., Gibson A.D., Mellanby R.J., Watson W., Appupillai M., Yale G., Gamble L. (2023). Community attitudes and perceptions towards free-roaming dogs in Goa, India. J. Appl. Anim. Welf. Sci..

[32] Macpherson C.N. (2012). Dogs, Zoonoses and Public Health.

[33] Coleman P.G., Dye C. (1996). Immunization coverage required to prevent outbreaks of dog rabies. Vaccine.

[34] Tiwari H.K., O’Dea M., Robertson I.D., Vanak A.T. (2019). Knowledge, attitudes and practices (KAP) towards rabies and free roaming dogs (FRD) in Panchkula district of north India: A cross-sectional study of urban residents. PLoS Negl. Trop. Dis..

[35] Tiwari H.K., O’Dea M., Robertson I.D., Vanak A.T. (2019). Knowledge, attitudes and practices (KAP) towards rabies and free-roaming dogs (FRD) in Shirsuphal village in western India: A community-based cross-sectional study. PLoS Negl. Trop. Dis..

[36] Singh B.B., Deka D.K., Kakati P. (2018). Epidemiology of Dog Bite Cases in India: A Systematic Review. J. Community Health.

