# Supplementary material for: Owned-Dog Demographics, Ownership Dynamics, and Attitudes across Three States of India
Source: Animals (Basel). 2024 May 14;14(10):1464. doi: 10.3390/ani14101464 (PMC11117354; doi:10.3390/ani14101464)
Supplement: Supplementary file 1 [file animals-14-01464-s001.zip › animals-2897376-supplementary.pdf]

Supplementary Material

Table S1. *Survey questions across Indian settlements. Each row represents a set of questions compared for the purposes of this study, with answer options given in italics. Note that not all questions are identical across settlements.*

Ahmedabad	Vadodara	Jamnagar	Nainital	Mussoorie	Kodaikanal	Coimbatore
**What is your gender?**						
- Male<br>*- Female*						
**Interviewer evaluation: Economic situation of the household**	**Interviewer evaluation: Type of Household**					
- Slum/Temporary Housing<br>*- Low Income Class*<br>*- Medium Income Class*<br>*- High Income Class*	*- Apartment*<br>*- Semi-detached House*<br>*- Detached House*					
**How many dogs do you own currently?**			**How many dogs do you own?**			**How many dogs do you own currently?**
Numeric						
**What is the sex of the dog?**				**Sex of the dog**		**What is the sex of the dog?**
- Male<br>*- Female*						
**Where did you get her/him from?**						
- Adopted (from shelter)<br>*- Adopted (from someone in neighbourhood)*<br>*- Adopted (from street)*<br>*- Bought (from in neighbourhood)*<br>*- Bought (from outside neighbourhood)*<br>*- Gift (from inside neighbourhood)*<br>*- Gift (from outside neighbourhood)*<br>*- Pup of my own dog*<br>*- Other/Don't know*						
**Why do you own dog(s)? (Multiple answers could be chosen)**						
- Pet/Companion<br>*- Breeding*<br>*- I want him/her to protect the property/crops*<br>*- Hunting*						
	**Why don't you own a dog?**					
	- I owned a dog but not currently<br>*- I don't like dogs*<br>*- It is against my religious beliefs*<br>*- No need for a dog*<br>*- No space for a dog*					
**Where is the dog at night?**						
- Free/roaming/loose outside<br>*- In the house*<br>*- Tethered outside*						
	**Was the dog vaccinated against rabies in the last 12 months?**		**Has your dog been vaccinated in the last 12 months?**		**Vaccinated in the last 12 months?**	**Was the dog vaccinated against rabies in the last 12 months?**

<table>
<tr><td></td><td colspan="6">- Yes
- No</td></tr>
<tr><td colspan="3">Is this dog sterilized?</td><td colspan="3">Is the dog neutered?</td><td>Is the dog sterilized?</td></tr>
<tr><td colspan="7">- Yes
- No</td></tr>
<tr><td>Why is your dog not sterilized?</td><td colspan="2">Why don't you want your dog to be sterilized?</td><td colspan="2">Why don't you want your dog to be neutered?</td><td></td><td>Why is your dog not sterilized?</td></tr>
<tr><td colspan="7">- Not necessary
- I don't have time
- Too expensive/I don't have the money
- I don't know where to have it sterilized
- I don't want my dog to become lazy
- I want my dog to protect my property
- I want to have puppies
- It is against my religious beliefs
- Too dangerous for the dogs
- I don't know</td></tr>
<tr><td colspan="4">Have you been to the veterinarian in the last 12 months?</td><td>Have you been to the vet in the last 12 months?</td><td>Have you been to the veterinarian in the last 12 months?</td><td rowspan="2"></td></tr>
<tr><td colspan="6">- Yes
- No</td></tr>
<tr><td colspan="7">Has anyone in the household been bitten by a dog in the last 12 months?</td></tr>
<tr><td colspan="7">- Yes
- No</td></tr>
<tr><td colspan="6">By which type of dog?</td><td>What type of dog was it?</td></tr>
<tr><td colspan="7">- Neighbour's dog
- Own household dog
- Unowned dog (in [settlement name])
- Unidentified strange dog</td></tr>
<tr><td>How many years have you been a dog owner?</td><td rowspan="4"></td><td rowspan="4"></td><td rowspan="4"></td><td rowspan="4"></td><td rowspan="4"></td><td rowspan="10"></td></tr>
<tr><td>- Numeric (years)</td></tr>
<tr><td>Is this (or one of them if more than one dog) the first dog you own?</td></tr>
<tr><td>- Yes
- No</td></tr>
<tr><td rowspan="2"></td><td colspan="2">Considering [the number of street dogs], what do you think about the number of dogs?</td><td colspan="3">What do you think about the number of dogs?</td></tr>
<tr><td colspan="5">- Far too few
- Too few
- Not too few and not too many
- Too many
- Far too many</td></tr>
<tr><td colspan="6">Street dogs are a danger to people where I live</td></tr>
<tr><td colspan="6">- Strongly agree
- Agree
- I don't know
- Disagree
- Strongly disagree</td></tr>
<tr><td colspan="6">Street dogs are a part of my community and are not a problem</td></tr>
<tr><td colspan="6">- Strongly agree
- Agree
- I don't know</td></tr>
</table>

<table>
<tr><td colspan="4">- Disagree
- Strongly disagree</td><td rowspan="9"></td></tr>
<tr><td colspan="3">How frequently (in a week's time) would you say you feel threatened by street dogs you meet on the streets?</td><td>How frequently would you say street dogs threaten you?</td></tr>
<tr><td colspan="4">- Never
- Rarely
- Sometimes
- Often
- Always</td></tr>
<tr><td rowspan="4"></td><td colspan="3">Do you think street dogs should be managed and if so how?</td></tr>
<tr><td colspan="3">- I don't know
- No, leave them alone / They are okay and don't bother anyone
- Sterilize, vaccinate and return them to the their street
- Remove, shelter and adopt them
- Euthanasia/killing/just take them all away</td></tr>
<tr><td>Street dogs should be removed</td><td>Street dogs should be removed from the streets</td><td>Street dogs should be removed</td></tr>
<tr><td colspan="3">- Strongly agree
- Agree
- I don't know
- Disagree
- Strongly disagree</td></tr>
<tr><td colspan="4">Do you feed street dogs?</td></tr>
<tr><td colspan="4">- Every day
- Once a week
- Several times a month
- Never</td></tr>
</table>

Table S2. *Census data for Indian settlements. Data drawn from: 2011 census (Government of India, 2011); * = estimates provided by municipalities at time of survey.*

Settlement	Survey Date	State	Settlement Type	Human Population	Households (HH)
Ahmedabad	Aug-19	Gujarat	Metro-Urban	7,322,403*	1,594,105*
Vadodara	Nov-17	Gujarat	Urban	1,752,371	376,276
Jamnagar	Oct-17	Gujarat	Semi-Urban	600,943	117,798
Coimbatore	Jul-17	Tamil Nadu	Urban	1,890,000*	282,839
Kodaikanal	Jun-17	Tamil Nadu	Semi-Urban	36,501	9,570
Mussoorie	Jul-17	Uttarakhand	Semi-Urban	30,118	6,245
Nainital	Jul-17	Uttarakhand	Semi-Urban	41,377	9,329

Table S3. *Count data of dog owning households by household type; sample sizes given in brackets.*

Settlement	N	Apartment	Semi-detached house	Detached house	Slum/ temporary housing	Low-income class	Middle-income class	Upper-income class
Ahmedabad	5633	-	-	-	6 (498)	42 (2000)	70 (2747)	12 (388)
Coimbatore	432	8 (75)	30 (168)	49 (189)	-	-	-	-
Jamnagar	409	4 (106)	11 (265)	4 (38)	-	-	-	-
Kodaikanal	369	3 (15)	17 (109)	69 (245)	-	-	-	-
Mussoorie	297	1 (7)	24 (156)	19 (134)	-	-	-	-
Nainital	328	5 (51)	44 (194)	13 (83)	-	-	-	-
Vadodara	2350	27 (620)	58 (1325)	45 (405)	-	-	-	-

Table S4. *Count data of dog acquisition sources. Question not asked in Coimbatore.*

Settlement	N	Adopted	Born to household	Bought	Received as a gift	Other
Ahmedabad	154	46	7	72	26	3
Coimbatore	NA	-	-	-	-	-
Jamnagar	20	1	0	10	9	0
Kodaikanal	101	17	18	41	23	2
Mussoorie	44	3	4	24	13	0
Nainital	69	38	0	20	11	0
Vadodara	136	20	10	78	28	0

Table S5. *Count data of reasons for dog ownership. Note that multiple responses could be given be each respondent. Question not asked in Coimbatore.*

Settlement	N	Pet/ companion	Property/ crop protection	Hunting	Breeding
Ahmedabad	132	124	15	0	1
Coimbatore	NA	-	-	-	-
Jamnagar	18	18	3	0	0
Kodaikanal	101	80	33	1	9
Mussoorie	44	26	28	0	1
Nainital	62	34	50	0	1
Vadodara	130	121	34	0	0

Table S6. *Count data of sterilisation, vaccination and vet visit counts for owned dogs. Sample sizes given in brackets. Vaccination status and vet visits were not investigated in Ahmedabad and Coimbatore respectively.*

Settlement	Sterilised	Sterilised (female)	Sterilised (male)	Vaccinated (last 12 months)	Vet visit (last 12 months)
Ahmedabad	52 (147)	23 (65)	29 (82)	-	119 (154)

Coimbatore	24 (121)	8 (37)	16 (83)	92 (122)	-
Jamnagar	1 (19)	1 (6)	0 (13)	15 (17)	7 (20)
Kodaikanal	20 (101)	5 (42)	15 (57)	66 (100)	57 (101)
Mussoorie	4 (28)	1 (5)	3 (23)	31 (39)	31 (44)
Nainital	30 (69)	16 (23)	14 (46)	52 (60)	52 (69)
Vadodara	50 (131)	11 (39)	39 (92)	126 (132)	102 (136)

Table S7. *Count data of dogs reported to be free roaming at night. Confinement practices were not investigated in Kodaikanal or Coimbatore.*

Settlement	N	Free roaming at night
Ahmedabad	154	13
Jamnagar	20	1
Mussoorie	21	0
Nainital	69	39
Vadodara	136	5

Table S8. *Count data of dog bite incidences concerning respondent or individuals within respondent's household in the last 12 months*

Settlement	N	Total bites	Own dog	Neighbour's dog	Unidentified/ unknown dog
Ahmedabad	5660	535	8	19	508
Coimbatore	432	16	4	3	9
Jamnagar	409	24	2	2	20
Kodaikanal	369	33	1	9	23
Mussoorie	297	14	0	7	7
Nainital	328	9	0	1	8
Vadodara	2350	145	8	14	123
